# Antrochoanal Polyps: Nasal Anatomical and Pathological Variants as Risk Factors

**DOI:** 10.1055/s-0046-1827782

**Published:** 2026-09-15

**Authors:** Ahmed Adel Sadek, Maisara Mohamed Mahmoud Nasr, Ahmed Ali Mohamed Badawi, Tamer El Zaeem Esmaeel, Osama Galal Awad

**Affiliations:** 1 Department of Otorhinolaryngology, Faculty of Medicine, Minia University, Minia, Egypt; 2 Department of Radiology, Faculty of Medicine, Minia University, Minia, Egypt

**Keywords:** nasal polyps, nasal septum, maxillary antrum, computed tomography

## Abstract

**Introduction:**

The etiopathogenesis of antrochoanal polyp remans debatable.

**Objectives:**

To understand antrochoanal polyp development and to assess anatomical and pathological risk factors for its formation.

**Methods:**

This study included 40 subjects with antrochoanal polyp as a study group and a control group of 40 subjects with unilateral maxillary sinusitis. We compared the groups with respect to pathologic variants including the presence of thickened mucosal lining and antral cyst in the contralateral maxillary sinus. We also compared groups regarding anatomic variants such as nasal septal deviation, accessory maxillary sinus ostium, concha bullosa, paradoxical middle turbinate, and uncinate process insertion.

**Results:**

Antral cyst was found in the contralateral maxillary sinus was significantly more prevalent in subjects with antrochoanal polyp than in subjects with unilateral maxillary sinusitis. Additionally, nasal septal deviation toward the opposite side of the pathology was significantly more frequent in antrochoanal polyp group than in unilateral maxillary sinusitis group.

**Conclusion:**

Antrochoanal polyp is suggested to originate from an antral cyst in the maxillary sinus, which extends into the nasal cavity due to pressure changes within the maxillary sinus and nasal cavity. Changes in the nasal cavity airflow caused by nasal septal deviation toward the opposite side of the affected maxillary antrum may represent an important risk factor for antrochoanal polyp formation from a pre-existing antral cyst.

## Introduction

Antrochoanal polyp (ACP) is among the most common unilateral sinonasal benign lesions. ACP is edematous mucosa that originates in the maxillary antrum. It enters the nasal cavity through the maxillary antrum ostium and then extends to the posterior nasal opening (choana). 1 The etiopathogenesis of ACP remains unknown. Several theories have been proposed to explain its pathogenesis. Some authors suggest that antral cyst of the maxillary sinus is the origin of ACP. 2 Others believe that nasal allergy and inflammation act as important factors in formation of edematous mucosa that progress to form ACP. 3 4 Nasal anatomical and pathological variants have been suggested as important risk factors for ACPs. These anatomical variations may serve as physical factor contributing to ACP formation.

## Methods

This prospective study was conducted at our Otolaryngology Department. The study was approved by the Institutional Review Board at our University. Approval No. 538–2022. The study was registered in ClinicalTrials.gov ID: NCT05989919. In this study, all participants were informed about the research and signed informed consent before data collection.

Subjects were recruited from patients attending the outpatient clinic.

Between December 2022 to September 2023, 40 patients with ACP were selected based on the following inclusion criteria: (1) radiological diagnosis based on CT of the nose and paranasal sinuses; and (2) the histopathological diagnosis of ACP after surgical excision. On CT, ACP appears as a low-attenuated soft tissue mass that fills the maxillary sinus cavity, extends into the nasal cavity through the middle meatus, and courses posteriorly toward the choana. Exclusion criteria: (1) history of previous nasal surgical intervention; and (2) history of maxillary or facial trauma.

A control group of 40 subjects with unilateral maxillary sinusitis attending our hospital during the same period, with the same exclusion criteria as the study group, was included.

Multi-slice computed tomography (CT) of nose and paranasal sinuses images was performed using a Toshiba Aquilion CT scanner. Coronal and axial sections with a slice thickness of 2.5mm were obtained.

The groups were compared with respect to anatomical and pathological variations in the nose. These variants include: nasal septal deviation, accessory maxillary sinus ostium, aerated middle turbinate (concha bullosa), paradoxical middle turbinate and insertion of uncinate process (to lamina papyrecea, ethmoid roof or middle turbinate). We also compared between groups according to presence of antral cyst and thickened mucosal lining in CT in maxillary sinus of the opposite side.

### Statistical Analysis

Statistical analyses were performed using SPSS software, version 24.0. Means and standard deviations were calculated for continuous variables. The Mann-Whitney U test and chi-square tests were used for between-group comparisons. Pearson correlation analysis was used to evaluate correlations between variables. Statistical significance was set at p ≤ 0.05.

## Results

The study included 80 subjects divided into two groups: the ACP group (n = 40) and the control group (n = 40). Table 1 shows the demographic data of the cases in each group. In ACP group, the mean age was 26.93 ± 11.92 years. There were 25 males and 15 females. In the control group, the mean is 27.78 ± 12.46 years, with 24 males and 16 females. There was no significant difference in age or sex between both groups ( *p* = 0.756 and p = 0.818, respectively).

**Table 1:** Demographic data

	Antrochoanal polyp group	unilateral maxillary sinusitis group	total	P value
Age Mean ± SD (Range)	26.93 ± 11.92 (8–50)	27.78 ± 12.46 (8–55)	27.35 ± 12.12 (8–55)	0.756
Males	25(62.5%)	24(60%)	49(61.2%)	0.818
Females	15(37.5%)	16(40%)	31(38.8%)	

**Fig. 1:**
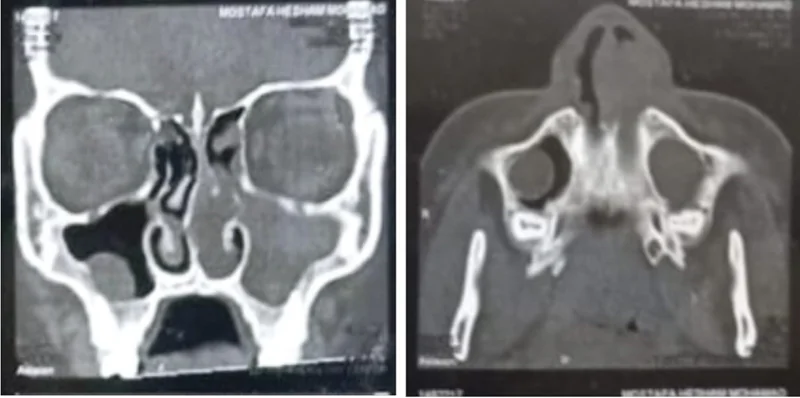
Axial and coronal CT nose and PNS showing left ACP and right antral cyst.

**Fig. 2:**
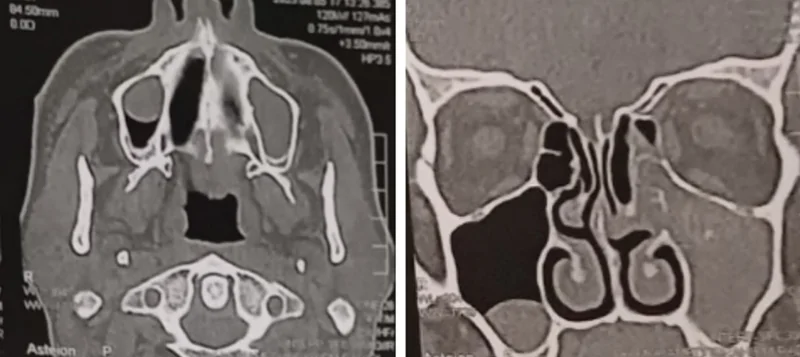
Axial and coronal CT nose and PNS showing left ACP, right antral cyst and deviated nasal septum to right side.

The prevalence of antral cyst in the contralateral maxillary sinus was significantly higher in the ACP group [15 cases (37.5%)] than in the unilateral maxillary sinusitis group [3 cases (7.5%)] (p = 0.001) ( Figs. 1 , 2 ).

The presence of thickened mucosa in the maxillary antrum did not differ significantly between the ACP group and the control group ( *p* = 0.396).

An accessory sinus ostium was present on the ipsilateral side of the pathology in 62.5% of ACP cases, through which the polyp protruded into the nasal cavity. In the remaining ASP cases, no accessory ostium was present, and the polyp extended through the natural ostium. This finding suggests that when an accessory ostium is present, the ACP invariably protrudes through it. No significant difference was observed between groups in relation to the presence of accessory maxillary sinus ostium on either the ipsilateral and contralateral side of the pathology ( *p* = 0.073, 0.117 respectively).

No statistically significant differences were found between the groups regarding concha bullosa or paradoxical middle turbinate, on either the ipsilateral or contralateral side of the pathology.

No statistically significant differences were found with respect to the superior insertion of uncinate process to lamina papyrecea, ethmoid roof or middle turbinate in both ipsilateral and contralateral side of the pathology. All of the previous results are shown in table 2 .

**Table 2:** Comparison of sino-nasal anatomic and pathologic variations between the ACP group & unilateral maxillary sinusitis group

	Antrochoanal polyp group	unilateral maxillary sinusitis group	P value
contralateral maxillary sinus antral cyst	15(37.5%)	3(7.5%)	0.001
contralateral maxillary sinus thickened mucosa	2(5%)	4(10%)	0.396
presence of accessory maxillary sinus ostium in the same side of the pathology	25(62.5%)	17(42.5%)	0.073
presence of accessory maxillary sinus ostium in the opposite side of the pathology	23(57.5%)	16(40%)	0.117
concha bullosa in the same side of the pathology	3(7.5%)	5(12.5%)	0.456
concha bullosa in the opposite side of the pathology	0(0%)	1(2.5%)	0.314
paradoxical middle turbinate in the same side of the pathology	3(7.5%)	4(10%)	0.692
paradoxical middle turbinate in the opposite side of the pathology	1(2.5%)	2(5%)	0.556
superior uncinate insertion to lamina papyrecea in the same side of the pathology	23(57.5%)	20(50%)	0.501
superior uncinate insertion to lamina papyrecea in the opposite side of the pathology	25(62.5%)	19(47.5%)	0.178
superior uncinate insertion to ethmoid roof in the same side of the pathology	14(35%)	12(30%)	0.633
superior uncinate insertion to ethmoid roof in the opposite side of the pathology	13(32.5%)	14(35%)	0.813
superior uncinate insertion to middle turbinate in the same side of the pathology	3(7.5%)	8(10%)	0.105
superior uncinate insertion to middle turbinate in the opposite side of the pathology	2(5%)	7(17.5%)	0.077
nasal septal deviation to same side of pathology	8(20%)	12(30%)	0.302
nasal septal deviation to opposite side of pathology	22(55%)	8(20%)	0.001

When the ACP group and the unilateral maxillary sinusitis group were compared with respect to nasal septal deviation as shown in table 2 , nasal septal deviation toward the contralateral side of the pathology was significantly more frequent in the ACP group [22 subjects (55%)] than in the unilateral maxillary sinusitis group [8 subjects (20%)] (p = 0.001) ( Fig. 2 ). Conversely, nasal septal deviation toward the ipsilateral side was present in 8 subjects (20%) in the ACP group and 12 subjects (30%) in the control group, a difference that was not statistically significant (p = 0.302).

## Discussion

**Fig. 3:**
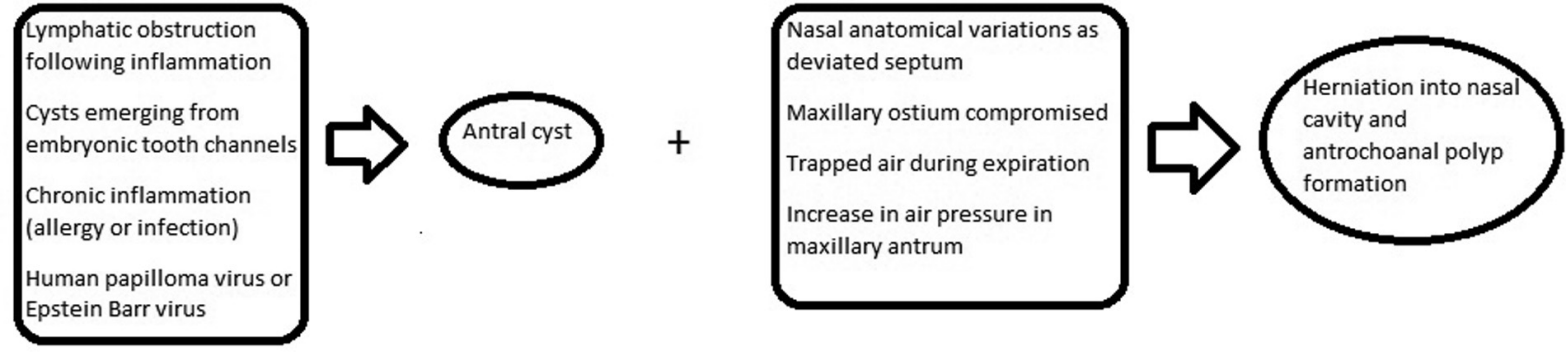
Summary of pathogenesis of antral cyst and antrochoanal polyp formation.

Although ACP has been recognized for a long time however, its pathophysiology and predisposing factors remain poorly understood. Numerous studies have been conducted to identify factors associated with ACP formation. Many hypotheses have been reported. One of these theories is the maxillary antrum cyst as an origin for antrochoanal polyp. Many suggestions were made to explain antral cyst formation. Chen et al. 5 reports that antral cysts form due to obstruction of acinar mucous glands by inflammation. Piquet et al. 6 proposed that antral cysts arise from an inflammatory process of the maxillary sinus mucosal lining. Some authors have proposed that antral cyst originate from intramural cysts arising from tooth channels. 3 7 Fig. 3 summarizes the possible theories for antral cyst formation then antrochoanal polyp formation. 8 9

Berg et al. 2 reported thar antral cyst is the origin of ACP, based on the histological similarity between ACP and antral cysts. In our study, the presence of antral cyst in the maxillary sinus of the opposite side was significantly higher in ACP patients than in unilateral maxillary sinusitis patients ( *p* = 0.001). This supports that ACP may have been a retention cyst originally. We hypothesize that retention cysts may be present in both maxillary sinuses, and that in the presence of changes in airflow and pressure within the maxillary antrum and nasal cavity, the antral cyst enlarges and protrudes into the nasal cavity through the middle meatus, ultimately forming ACP. Anatomic variants have a significant role in air flow & pressure changes in the nasal cavity. This remains in hypothesis, and longitudinal follow-up on antral cysts would be required to confirm their potential transformation into ACP. This is practically challenging, as patients with antral cysts are typically asymptomatic.

Frosini et al. 8 explain this mechanism according to the Bernoulli theorem. This theorem states that airflow passing through a narrow area increases in velocity and decreases in pressure. During inspiration, airflow velocity increases, according to this principle, the associated pressure decrease may lead to collapse of the nasal walls. During expiration, air outflow from the maxillary ostium decreases, causing increased intrasinus pressure that may result in herniation of the polyp. Frosini et al also suggested that there was narrowing or obstruction of the natural maxillary sinus ostium which may be due to inflammatory process, so the ACP protrudes though the accessory ostium. 8 In our study, whenever there is accessory ostium, all the ACP get through it. The presence of antrochoanal polyp unilaterally is due to lack of negative pressure in the nasal cavity of the opposite side which may be due to anatomical variants as deviation of nasal septum or because of antrochoanal polyp occupying the nasopharynx, so there is less negative pressure to pull the maxillary contents into the cavity of the nose. 10

Presence of inflammation leads to narrowing of the maxillary ostium, thus aggravates the condition. Gursoy et al. 11 suggested that chronic inflammation is an important factor for ACP formation. The authors stated that maxillary sinus sclerosis and thickened mucosa are indicators of a chronic inflammatory process. In their study, the presence of maxillary sinus wall sclerosis was significantly higher in ACP patients than in unilateral maxillary sinusitis patients ( *p* = 0.001). However, in our study, the presence of sinus wall thickened mucosa wasn't significantly different between ACP patients and unilateral maxillary sinusitis patients.

When we compared the presence of deviated nasal septum between both groups in this study, we found 22(55%) patients with nasal septal deviation to contralateral side of pathology in ACP patients [ Figure 2 ] and 8 (20%) cases in the control group and that was statistically significant ( *p* = 0.001). Hekmatnia et al. 12 reported a higher frequency of nasal septal deviation in ACP patients. Aydin et al. 13 observed that the nasal septum was deviated toward to the contralateral side of the ACP, suggesting that this occurs true a mechanism similar to compensatory hypertrophy of the inferior turbinate on the side opposite to the nasal septal deviation. Elahi et al. 14 said reported that ACP developed on the concave side of the deviated nasal septum, owing to associated middle turbinate abnormalities on the side opposite to the deviation. Unfortunately, it cannot be determined with certainty whether the antrochoanal polyp led to contralateral nasal septal deviation, or whether the septum was already deviated prior to ACP development and the affected air flow in the nasal cavity has led to ACP formation. This limitation is due to inherent challenges in monitoring ACP formation from its onset.

In the study done by Gursoy et al, the incidence of the presence of maxillary sinus accessory ostium in the ipsilateral side of ACP group was significantly high (72.9%). 11 In our study it was found in 62.5% but it was statistically insignificant when it was compared with the control group ( *p* = 0.073).

Other anatomic variants including: paradoxical middle turbinate, concha bullosa and variation in superior insertion of uncinate was statistically insignificant when we compared ACP group and control group. This agrees with the study done by Gursoy et al. 11

In this study, the male-to-female ratio is 1.6:1, other studies done by Aktaş et al & Bozzo et al showed higher incidence in males. 15 16

Further studies employing objective methods to measure nasal airflow resistance and pressure changes are needed to support these findings.

## Conclusion

Although antrochoanal polyp is among the most common as a unilateral maxillary antrum lesions, its pathogenesis remains unclear. The present findings support the hypothesis that the precursor of ACP is an antral cyst which extends into the nasal cavity as a result of pressure changes in the maxillary sinus and nasal cavity. Changes in nasal cavity airflow caused by nasal septum deviation toward the contralateral side may represent an important risk factor contributing to ACP formation from a already pre-existing antral Cyst.

## References

[1] Killian G The origin of choanal polypi Lancet 1906 168(4324):81 82

[2] Berg O Carenfelt C Silfverswärd C Sobin A Origin of the choanal polyp Arch Otolaryngol Head Neck Surg 1988 114 11 1270 1271 3166756 10.1001/archotol.1988.01860230064025

[3] Cook P R Davis W E McDonald R McKinsey J P Antrochoanal polyposis: a review of 33 cases Ear Nose Throat J 1993 72 06 401 402, 404–410 8344181

[4] Lee T-J Huang S-F Endoscopic sinus surgery for antrochoanal polyps in children Otolaryngol Head Neck Surg 2006 135 05 688 692 17071295 10.1016/j.otohns.2006.02.035

[5] Chen J M Schloss M D Azouz M E Antro-choanal polyp: a 10-year retrospective study in the pediatric population with a review of the literature J Otolaryngol 1989 18 04 168 172 2661853

[6] Piquet J J Chevalier D Leger G P Rouquette I Leconte-Houcke M [Endonasal microsurgery of antro-choanal polyps] Acta Otorhinolaryngol Belg 1992 46 03 267 271 1414308

[7] Berg O Carenfelt C Sobin A On the diagnosis and pathogenesis of intramural maxillary cysts Acta Otolaryngol 1989 108(5-6):464 468 2589074 10.3109/00016488909125554

[8] Frosini P Picarella G De Campora E Antrochoanal polyp: analysis of 200 cases Acta Otorhinolaryngol Ital 2009 29 01 21 26 19609378 PMC2689564

[9] Almarri F K AlHumaizi A Alomair A M Bilateral antrochoanal polyp: a case report of an extremely rare entity managed conservatively with a review from the past 26 years Ann Med Surg (Lond) 2023 85 03 611 617 37008176 10.1097/MS9.0000000000000290 PMC10060084

[10] Al-Qudah M Bilateral antrochoanal polyps: possible pathogenesis J Craniofac Surg 2011 22 03 1116 1118 21586961 10.1097/SCS.0b013e3182108f0a

[11] Gursoy M Erdogan N Cetinoglu Y K Dag F Eren E Uluc M E Anatomic variations associated with antrochoanal polyps Niger J Clin Pract 2019 22 05 603 608 31089013 10.4103/njcp.njcp_419_18

[12] Hekmatnia A Shirvani F Mahmoodi F Hashemi M Association of anatomic variations with antrochoanal polyps in paranasal sinus computed tomography scanJ Res Med Sci Off J Isfahan Univ Med Sci2017 10.4103/1735-1995.199085 PMC5361439 28400825

[13] Aydın S Taskin U Orhan I The analysis of the maxillary sinus volumes and the nasal septal deviation in patients with antrochoanal polyps Eur Arch Otorhinolaryngol 2015 272 11 3347 3352 25534286 10.1007/s00405-014-3460-1

[14] Elahi M M Frenkiel S Fageeh N Paraseptal structural changes and chronic sinus disease in relation to the deviated septum J Otolaryngol 1997 26 04 236 240 9263892

[15] Aktaş D Yetişer S Gerek M Kurnaz A Can C Kahramanyol M Antrochoanal polyps: analysis of 16 cases Rhinology 1998 36 02 81 85 9695164

[16] Bozzo C Garrel R Meloni F Stomeo F Crampette L Endoscopic treatment of antrochoanal polyps Eur Arch Otorhinolaryngol 2007 264 02 145 150 17013627 10.1007/s00405-006-0175-y

